# Increasing peptide identifications and decreasing search times for ETD spectra by pre-processing and calculation of parent precursor charge

**DOI:** 10.1186/1477-5956-10-8

**Published:** 2012-02-09

**Authors:** Viswanadham Sridhara, Dina L Bai, An Chi, Jeffrey Shabanowitz, Donald F Hunt, Stephen H Bryant, Lewis Y Geer

**Affiliations:** 1 National Library of Medicine, NIH, Bethesda, Maryland, USA; 2 Department of Chemistry, University of Virginia, Charlottesville, Virginia, USA; 3 Merck Research Laboratories, Boston, Massachusetts, USA; 4 Department of Pathology, University of Virginia, Charlottesville, Virginia, USA

## Abstract

**Background:**

Electron Transfer Dissociation [ETD] can dissociate multiply charged precursor polypeptides, providing extensive peptide backbone cleavage. ETD spectra contain charge reduced precursor peaks, usually of high intensity, and whose pattern is dependent on its parent precursor charge. These charge reduced precursor peaks and associated neutral loss peaks should be removed before these spectra are searched for peptide identifications. ETD spectra can also contain ion-types other than c and z**˙**. Modifying search strategies to accommodate these ion-types may aid in increased peptide identifications. Additionally, if the precursor mass is measured using a lower resolution instrument such as a linear ion trap, the charge of the precursor is often not known, reducing sensitivity and increasing search times. We implemented algorithms to remove these precursor peaks, accommodate new ion-types in noise filtering routine in OMSSA and to estimate any unknown precursor charge, using Linear Discriminant Analysis [LDA].

**Results:**

Spectral pre-processing to remove precursor peaks and their associated neutral losses prior to protein sequence library searches resulted in a 9.8% increase in peptide identifications at a 1% False Discovery Rate [FDR] compared to previous OMSSA filter. Modifications to the OMSSA noise filter to accommodate various ion-types resulted in a further 4.2% increase in peptide identifications at 1% FDR. Moreover, ETD spectra when searched with charge states obtained from the precursor charge determination algorithm is shown to be up to 3.5 times faster than the general range search method, with a minor 3.8% increase in sensitivity.

**Conclusion:**

Overall, there is an 18.8% increase in peptide identifications at 1% FDR by incorporating the new precursor filter, noise filter and by using the charge determination algorithm, when compared to previous versions of OMSSA.

## Background

Mass-spectrometry based proteomics is a major technique for the identification of the constituents of complex protein mixtures [1]. Analysis of peptide and protein sequences using gas-phase ion chemistry and tandem mass spectrometry has been described by various groups [2-5], where common methods of peptide identification involve enzymatic digestion of proteins isolated from protein mixture, fractionation and fragmentation of the resultant peptides, followed by MS/MS sequence search algorithms to match the peptide sequence to the tandem mass spectrometry data. Some of the widely used search algorithms are OMSSA [6], X!Tandem [7], Sequest [8], MyriMatch [9], SpectrumMill (Agilent) and Mascot [10]. A key step is the fragmentation method used to dissociate the peptides obtained after enzyme cleavage. Techniques currently used include Collision Activated Dissociation [CAD], Electron Capture Dissociation [ECD] and ETD [11]. The use of ETD is becoming increasingly prevalent as it can be used on more common instruments such as quadrupole ion trap.

ETD/ECD spectra can contain precursor peaks in various charge states, called charge reduced precursors. Neutral losses from these precursors are shown to be prevalent among both ECD [12] and ETD spectra [13]. Some of the widely observed neutral losses in ETD are ammonia (17 Da), water (18 Da), and carbon monoxide (28 Da). Also, the presence of arginine in the peptide sometimes leads to loss of a guanidino group (43 Da). In an effort to reduce false positives and improve sensitivity (true positives/total hits) in OMSSA, the intense precursor and neutral loss peaks that can be present in ETD MS/MS spectra should be removed before these spectra are searched against the protein sequence library. OMSSA currently has a precursor filter [14] which removes these precursor peaks and neutral losses. We employed modifications to this present routine to accommodate higher charged precursor peaks and their associated neutral losses.

**Figure 1:**
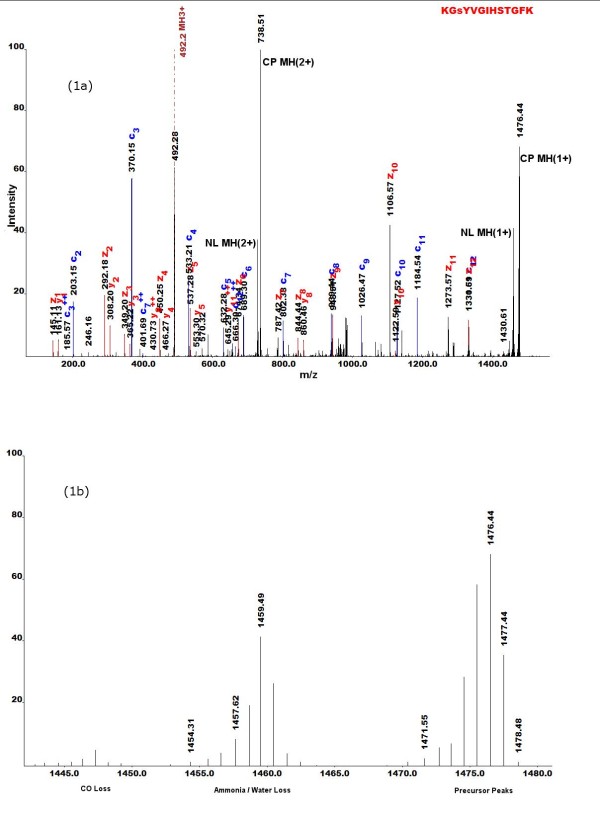
**ETD MS/MS spectra**. a. Charge reduced precursors (CP) and neutral losses (NL), along with fragment ion peaks are shown for peptide KGsYVGIHSTGFK. The first serine is phosphorylated in this peptide and hence is shown in lower case. b. Precursor peaks and their associated neutral losses are shown here. Removing these intense precursor and neutral loss peaks would aid in improving the sensitivity of sequence search algorithms.

ETD spectra can contain multiple ion types [13,15,16]. These studies have shown the occurrence of y ions along with that of the more common c and z**˙ **ions in the ETD MS/MS spectra. Protein sequence library search algorithms should consider these ion-types in their noise filtering and scoring algorithms. Here, we accommodated such changes in OMSSA's noise filter. Figure 1 shows c, z**˙ **and y ions present in an ETD MS/MS spectra.

ETD works exceptionally well on large multiply charged peptides [17]. In the MS/MS dataset used in this paper the precursor charge ranges from 3+ to 7+. Unlike high resolution instruments, lower resolution instruments, such as the linear ion trap used in this experiment, are typically not used to determine a precursor charge. Peptide identification is generally done by MS/MS database search algorithms, which generally require either the precursor charge or a possible range as input along with the precursor mass and the peak list file. If the range option is used, the algorithm looks for peptides with different possible precursor charge range and molecular weights, which can be computationally expensive and result in false positives. Determining the precursor charge state accurately may improve sensitivity and specificity along with the sequence library search times. Several algorithms have been developed [18-20] to determine the parent precursor charge from the tandem mass spectrometry data. Charger [18] uses 2 methods to infer precursor charge state. The first method employs self-correlation analysis of product ions to infer precursor charge from the peptide mass, obtained from the complementary ions. When the 1^st ^method fails, Charger uses linear discriminant analysis [LDA] to predict charge states using different features of the ETD spectra. The Charge Prediction Machine [19] employs Bayesian decision theory to classify charge states using the features found in the ETD spectra. Recently, another algorithm to determine the precursor charge using support vector machine [SVM] has been developed [20]. The algorithm described in this paper uses LDA with a unique set of features to estimate if a charge state can be assigned to a spectrum, and if so, what charge states are possible. The algorithm is trained not to assign a charge state if the features do not warrant determination of the precursor charge.

## Results and discussion

In this study, we examined algorithms for pre-processing and charge determination of ETD spectra. To validate these algorithms, we used a dataset consisting of ETD MS/MS spectra of yeast phosphopeptides [21]. The dataset has a total of 16901 spectra, of which 10000 spectra were used as training set, while the rest were used as test set. We searched the spectra against a target-decoy library [22] using target sequences from the NCBI 6298 yeast protein sequence library.

### Spectral pre-processing

Spectral pre-processing prior to protein sequence library search is an important step to identify high-confidence peptide-spectrum matches. Generally, this pre-processing step involves removing possible noise peaks and other non-product ion peaks, for example, precursor peaks and their associated neutral losses in ETD spectra. These pre-processing steps, which are already present in OMSSA [14], were revised to accommodate more ion-types and higher precursor charge states. For our analysis here, we divided this spectral pre-processing step into 2 stages of filtering -- precursor filtering and noise filtering.

#### Precursor filter

Other than the product ion fragmentation, MS/MS spectra obtained from ETD can contain charge reduced precursor peaks, usually of high intensity. This series of charge reduced precursor peaks is distributed in n non-overlapping bins of the MS/MS spectra where n is the parent precursor charge. These bins are mass windows around <MH(z+)>, where z ranges from 1 to n, M is neutral mass of the peptide, H is mass of proton and MH(z+)=(M+zH)/z. Figure 1b illustrates the precursor peaks and their associated neutral losses observed in an ETD spectrum. The width of the isotopic distribution of these ions generally depends on their mass.

We looked at various options to remove these precursors and their neutral losses, e.g.

(a) removal of a fixed window width around these precursor peaks,

(b) removal of a *variable* window width around these precursor peaks, and

(c) removal of the neutral loss region (-60 Da or -18 Da, scaled to z) for the precursor and its reduced series.

The motivation behind using a variable window is to make the algorithm applicable for both smaller peptides (3+ parent charge) and larger peptides (7+ or higher charged precursor peptides). If we use a "fixed" window around the precursor, we may remove "product ion signal" regions for lower charged precursors (3+) or in case of higher charged precursors (7+), we may retain these precursor peaks, which could affect the scoring adversely. We compared our results with a recently developed spectral processing algorithm by Good et al [23] and the previous OMSSA filter. Below is some terminology used in this comparision:

W→ Window upstream of monoisotopic precursor peaks,

M→ Neutral mass of peptide,

n→ Parent precursor charge,

N1, N2→ Width of neutral losses which are downstream to precursor peaks,

H→ Mass of proton and

mz→ Corresponds to the region where all the peaks are removed.

##### Removal of precursor peaks

(1) $$\text{Good}\;\text{et}.\;\text{al}.^{\prime }\text{s}\;\text{Algorithm}:\text{MH}\left(\text{z}+\right)-3.1<\text{mz}<\text{MH}\left(\text{z}+\right)+3.1,\text{for}\;\text{z}=1\;\text{to}\;\text{n}.$$

(2) $$\text{OMSSA}:\text{MH}\left(\text{z}+\right)<\text{mz}<\text{MH}\left(\text{z}+\right)+\text{M}/\left(\text{W}*\text{z}\right),\;\text{for z}=1\text{ to n}.$$

##### Removal of neutral loss peaks

(3) $$\text{Good et}.\text{ al}.^{\prime }\text{s Algorithm}:\text{MH}\left(\text{z}+\right)-\text{N}1/\text{z}<\text{mz}<\text{MH}\left(\text{z}+\right),\text{ for z}=1\text{ to n}.$$

(4) $$\text{OMSSA}:\text{MH}\left(\text{z}+\right)-\text{N}1/\text{z}<\text{mz}<\text{MH}\left(\text{z}+\right),\text{ for z}=1\text{ and }2,$$

(5) $$\text{MH}\left(\text{z}+\right)-\text{N}2/\text{z}<\text{mz}<\text{MH}\left(\text{z}+\right),\text{ for z}=3\text{ to n}.$$

W, N1 and N2 are set to 500, 60 Da and 18 Da respectively. We use these values for these variables as they gave good performance with the training set. For removal of neutral losses accompanying MH(1+) and MH(2+) precursors, we removed all the peaks which fall below 60 Da and 30 Da respectively from the precursor peak. Similar reasoning has been employed by Sweet et. al.,[24], but they did not consider a large neutral loss window for MH(2+). In our analyses, we changed the bin sizes around the precursor peaks (W, N1 and N2 in equations above) to optimize sensitivity and specificity for the training set. Combining (2), (4) & (5), we get the overall equations to remove the precursor peaks and their associated neutral losses in OMSSA spectral precursor filtering:

(6) $$\text{OMSSA}:\text{MH}\left(\text{z}+\right)-\text{N}1/\text{z}<\text{mz}<\text{MH}\left(\text{z}+\right)+\text{M}/\left(\text{W}*\text{z}\right),\text{ for z}=1\text{ and }2,$$

(7) $$\text{MH}\left(\text{z}+\right)-\text{N}2/\text{z}<\text{mz}<\text{MH}\left(\text{z}+\right)+\text{M}/\left(\text{W}*\text{z}\right),\text{ for z}=3\text{ to n}.$$

**Figure 2:**
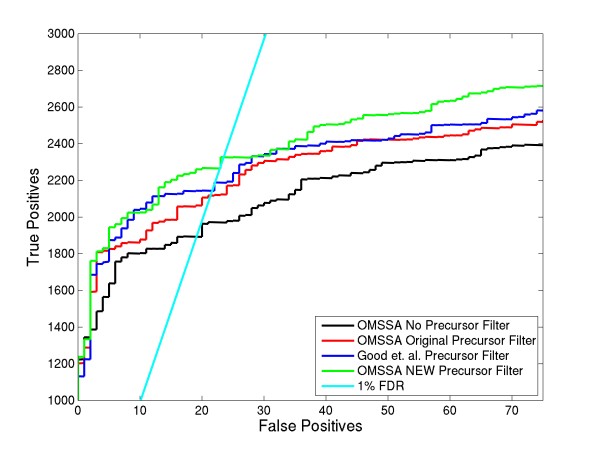
**Comparison of different variants of OMSSA precursor filter**. This figure compares the number of peptide identifications using Good et al.'s algorithm and the OMSSA algorithm using the new and older versions of precursor filter.

To validate the algorithm, we did OMSSA searches on the test set using the previous precursor filter in OMSSA, the newer precursor filter, the Good et. al. precursor filter and also using the "no precursor filter" option in OMSSA. We then compared the sensitivity and specificity of the peptide identifications using these methods by looking at the ROC curves. Results of ROC analysis is shown in Figure 2. A 1% FDR line is drawn to show the differences in peptide identifications among the various searches. At 1% FDR, OMSSA's older version of precursor filter identified 2118 true positives, while Good et. al.'s filter identified 2143 true positives. When the new precursor filter is introduced into OMSSA, there were 2326 true positives, i.e., an increase of ~8.5% in peptide identifications compared to Good et. al.'s algorithm. Using OMSSA with "no precursor filter" option, we found only 1893 true positives, clearly showing that removal of these precursor peaks and neutral losses results in increased peptide identifications. We incorporated the new precursor filter routine in OMSSA for all other analysis described hereafter.

#### Noise filter

Apart from the precursor filtering routine, OMSSA employs a noise filtering routine to remove noise peaks found in the mass spectra prior to submitting the spectra to sequence library search. In this noise filtering algorithm, first step involves removing the isotope peaks, other than the monoisotopic product ion-peak. This is done by removing peaks which are 1-2 Da upstream of the most intense peak. We did not make any changes to this routine. The second step in this noise filtering involves removing peaks that are too close together. This is explained in detail in the original OMSSA paper [6]. This filter retains the top 2 most intense peaks in a sliding window of +/- 27 Da (or +/- 14 Da) when looking for 1+ (or 2+) product ion peaks. The reason to pick 2 peaks is that the filter assumes there is one forward ion series (c ions) and one reverse ion series (z**˙) **in each region. Since it has been shown that ETD spectra can also contain other ion types [13,15,16], we modified this routine to accommodate for extra ion-types present in the spectra. For example, if we are looking for c, z**˙ **and y ions, then the probability of any product ion being found in a +/- 27 Da window would be more than if we are looking for only c and z**˙ **ions. In OMSSA, the number of 1+ product ion peaks allowed in a window of +/- 27 Da is given by "h1", while the number of 2+ product ion peaks allowed in a +/- 14 Da window is given by "h2". By default, these values are set to 2. Since we are using c, z**˙ **and y ions in our peptide sequence library search, we ran OMSSA searches on the training set to find that the optimum value for "h1" and "h2" is 3. Hence, we made "h1" and "h2" equal to the number of possible ions found in the spectra. OMSSA users can change these values, if needed.

**Figure 3:**
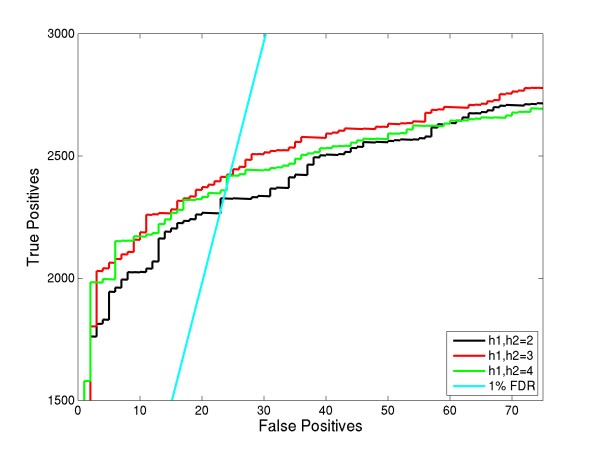
**OMSSA noise filter improvements**. This figure compares the different options in OMSSA noise filter (h1 and h2) for spectra searched for 3 ion species. By default, OMSSA sets a value of 2 for these parameters. Looking at the ROC plot, we modified these parameters "h1" and "h2" to be equal to the number of ions searched for, to yield higher sensitivity without loss in specificity.

We ran OMSSA searches on test set to understand the differences caused by varying "h1" and "h2". For this, we considered 3 cases: h1, h2 = 2 (default value), h1, h2 = 3 (new noise filter) and also h1, h2 = 4. ROC curves for these searches is shown in Figure 3. At 1% FDR, using the previous version of noise filter (h1, h2 = 2), there were 2326 true positives. Using, h1, h2 = 3, we found that OMSSA identified 2423 true positives, a 4.2% increase in peptide to spectrum identifications when compared to the previous version of the noise filter. For h1, h2 = 4, clearly there was a loss of peptide identifications, which shows that the optimum value for the number of peaks in a sliding window (i.e., h1 and h2 in OMSSA) should be equal to the number of ion-types searched for (here c, z**˙ **and y ions). If the spectra are found to contain only 2 ion-types, then h1 = h2 = 2 is an optimum value. These results show the importance of spectral pre-processing in increasing the number of peptide identifications at a given FDR. We have incorporated this noise filter in OMSSA and make a general recommendation to include y-ion in ETD searches.

### Precursor charge determination

#### Charge reduced precursor series

Feature selection is one of the important methods in any classification problem. There are several features in ETD MS/MS spectra that can be used to deduce precursor charge. Some of the more important ones are charge reduced precursor peaks and neutral losses. Figure 1 shows a MS/MS scan of a peptide KGsYVGIHSTGFK with charge reduced precursor peaks and their associated neutral losses. Manual validation of this spectrum shows that the parent precursor charge is +3 with a precursor mass of 492.2 m/z (MH 3+). For this ion, other charge reduced non-dissociated precursor series include the peaks at 1474.6 m/z (MH 1+) and 737.8 m/z (MH 2+). These m/z values match well with the intense peaks found in the spectra in Figure 1. If the same precursor mass was assumed to have precursor charge of 4+, then charge reduced non-dissociated precursor series include peaks at 1965.8 m/z (MH 1+), 983.4 m/z (MH 2+) and 655.9 m/z (MH 3+). These peaks do not overlap with the +3 charged reduced precursors. The precursor charge can be correctly identified using the correct precursor mass from the experiment and comparing it to the charge reduced peaks in the spectra. Since we need to extract precursor peak information from the MS/MS spectra as input to the LDA, we used the following equation to represent this feature:

(8) $$\text{MH}\left(\text{z}+\right)-\text{tolp}/\text{z}<\text{mz}<\text{MH}\left(\text{z}+\right)+\text{X}\left(\text{M},\text{z}\right)+\text{tolp}/\text{z},\text{ z}=1\text{ to n}.$$

where X(M, z) = M/(W*z) and W = 500. Parameter W is taken from the precursor filter analysis. If M is 2000 Da, then X for MH(2+) will be 2000/(500*2) = 2 Da. Similarly for MH(1+), X will be 4 Da. Parameter tolp is set to 2 Da.

Similarly, neutral losses associated with these precursor peaks can be calculated, depending on the types of neutral losses, which is explained below in detail.

#### Neutral losses

There are scenarios where two precursors with different charge states can have overlapping charge reduced precursor bins. A 2+ precursor has overlapping charge reduced precursor peaks with those of 4+, 6+ and other multiples of 2+. Similarly a 3+ precursor will have overlapping charge reduced precursor peaks with 6+, 9+ and so on, as 6+, 9+ etc. are multiples of 3+. Identifying charge state in these cases is sometimes ambiguous. Considering neutral loss peaks may circumvent this problem of identifying multiples. For example, a charge reduced precursor mass of 300 Da, with precursor charge of +2 will have MH+ precursor peaks around bin centered at 599 Da, assuming a proton mass of 1 Da for simplification. Similarly, a 4+ precursor charge will have MH(2+) precursor peaks around the similar bin at 599 Da too. If we consider neutral losses (for example, ammonia and water losses) along with the peaks of reduced precursor series, then for a 2+ precursor charge with 300 m/z precursor mass, neutral losses of the precursor peak MH+ are centered at 18 Da from precursor peaks at 599 Da, which is approx. 581 Da. On the other hand, for 300 m/z precursor mass with 4+ charge, MH(2+) neutral losses are 9 Da away from its precursor peaks at 599 Da, which is approx. centered at bins around 590 Da, different than 581 Da for 2+ precursor charge. So, the presence of neutral loss peaks at bin centered at 590 Da suggests there is a higher chance that the precursor could be of 4+ charge, rather than a 2+. Taking into account the neutral losses as a feature may improve the sensitivity of the charge determination algorithm. We have considered neutral losses of water and ammonia, which are -18 Da and -17 Da (scaled to z) respectively from their precursor peaks.

(9) $$\text{MH}\left(\text{z}+\right)-\text{N}2/\text{z}-\text{tol}/\text{z}<\text{mz}<\text{MH}\left(\text{z}+\right)-\text{N}2/\text{z}+\text{tol}/\text{z},\text{ if z}=1\text{ to n}$$

where tol is the tolerance (bin width) around the neutral losses window and N2 is the neutral loss considered in this analysis. The values considered for N2 and tol are 18 Da and 4 Da respectively. One of the differences between this equation compared to (4) and (5) is that here we removed only neutral loss peaks of water and ammonia, instead of removing the entire region from the precursor to these peaks, the reason being the identification of the precursor charge exactly rather than spectral cleaning. All the features used in this study are normalized with the total ion current present in the spectra. OMSSA searches with assigned precursor charges from the algorithm and by general range search method are compared using ROC curves and the results are described below.

For the charge determination algorithm, we used the same training and test sets that we used for the spectral pre-processing. There were some differences on how we used the training set to build a LDA classifier. Our first step in this analysis is to find a good set of peptide-spectrum matches to classify the spectra into different charge states based on the information obtained from the MS/MS scan. We used OMSSA on the training set to get peptide-spectrum matches. To have a reliable set to input LDA, we used an OMSSA e-value cut-off of 1e-6 on the training set results to pick high-confidence peptide-spectrum matches. We chose this e-value to avoid any decoy assignments. In cases where OMSSA results in identification of 2 peptides (for a ETD MS/MS spectrum) with different precursor charges, we used the top-most hit. We found 428 unique peptide-spectrum matches that satisfy these criteria and are used as input to LDA classifier.

Two features of interest i.e., charge reduced precursor peaks and their associated neutral losses are extracted from the spectra, as described in equations 8 and 9. These are then used as inputs to the LDA. The output of this LDA classifier is the best predicted charge state and the posterior probabilities of each of the charge states. These posterior probabilities could be used to find the ascending order of predicted precursor charge states. In cases such as mixture spectra, which contain peaks generated by two peptides that could have different precursor states, considering the top 2 possible charge states predicted by LDA is helpful, and in cases when there is not enough information obtained from the ETD MS/MS scan, range search is considered. We considered the following 3 scenarios:

1. Top 1: Only the best predicted charge state is used to search for peptides using OMSSA.

2. Top 1/Top 2: Consider a threshold t1 for the posterior probabilities obtained from LDA classifier and then assign the top 2 predicted charges to the spectra whose posterior probability of the best predicted charge is below t1, while considering only the best predicted charge for spectra above this threshold t1. This can be considered similar to Charger [18], as it can assign 2 best predicted charge states if it cannot assign a single best possible precursor charge.

3. 1/2/All: A third scenario is considered, where the 2 thresholds t1 and t2 are considered. If the posterior probability of the best predicted charge is greater than t1, only the best charge is considered. While if the probability falls between t1 and t2 (t1>t2), then top 2 charges are searched. If the probability is less than t2, the spectra is assigned the entire range to search for the probable precursor charge states. This is similar to changing the relaxation parameter in the Charge Prediction Machine [19]. We determined the thresholds using the training set. For the present analysis, we found that optimum values for t1 and t2 are 0.99 and 0.9 respectively. We varied the settings for t1 and t2 and selected the values that worked best. Introducing few false positives, rather than losing many true positives, is one of the criteria in determining the above threshold values.

**Figure 4:**
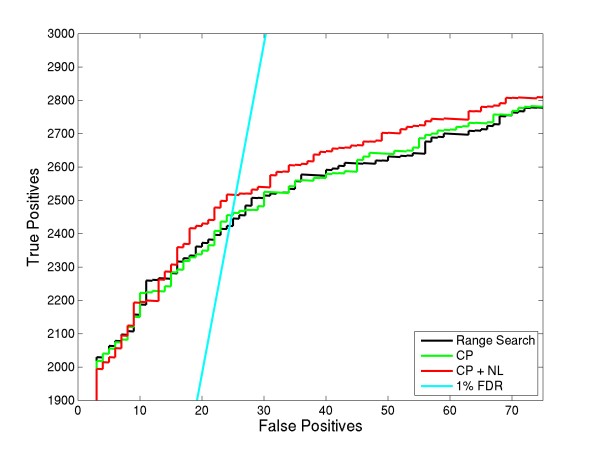
**Comparison of different input LDA features for the precursor charge determination algorithm**. This plot compares the differences between choosing input features for LDA. CP represents charge reduced precursor peaks and NL represents neutral losses. As a baseline, ROC curve using the OMSSA's range search is also provided. Using CP and NL as features shows an increase in peptide identifications over the range search method.

To optimize features and thresholds, we compared ROC plots. For the first analysis, we considered various combinations of input features used in LDA and then compared these results to the generally employed range search method, which examines the entire possible charge range (+3 to +7 here) for all of the spectra. In the 1st OMSSA run, we considered only the precursor peaks (CP) as the input features. For the 2nd run, we considered both the precursor peaks and the neutral losses (CP+NL) as the input features. For all these runs, we considered the 1/2/All variant of the LDA charge assignment. In other words, we assigned the top charge found by LDA for spectra above a threshold t1, then assigned the top 2 charges to spectra below t1 but above t2 (t1>t2) and assigned range search (+3 to +7, here) for the remaining spectra. Figure 4 shows the ROC plot of these OMSSA searches.

Using the precursor peaks (CP) alone as an input feature did not result in an improvement in peptide identifications compared to the general range search method, although it did result in a significant decrease in search time (see Table 1). Using both the precursor peaks and neutral losses as input features resulted in more identifications compared to the general range search method and also decreased search time. The increase in peptide identifications using both features compared to using only precursor peaks as feature could possibly be due to some spectra having intense neutral losses compared to precursor peaks or could be due to overlapping charge reduced precursors. In the latter case, LDA classifier could not differentiate between multiples. At 1% FDR, CP + NL precursor charge assignment resulted in 2516 identifications, which is a 3.8% increase in identifications compared to the range search method.

**Table 1:** Table showing database search times for OMSSA using different variants of precursor charge determination algorithm.

Method	Computational Time (minutes)
Range Search	91

CP+NL (Top 1)	9

CP+NL (Top 1/Top 2)	17

CP+NL (1/2/All)	26

This table shows the computation time for range search and for variants of the precursor charge assignment algorithm. Database search times are faster if we consider only few charge states for the spectra.

**Figure 5:**
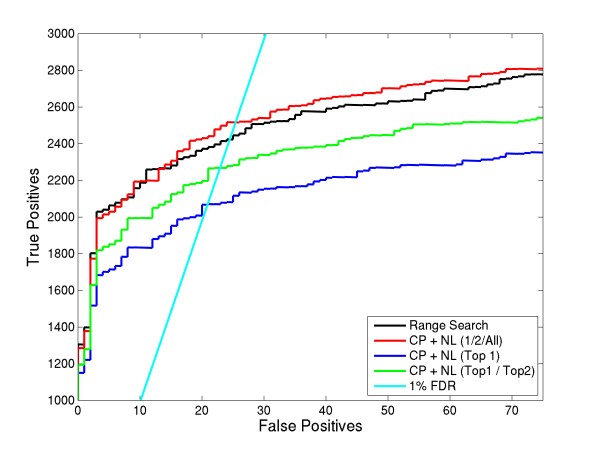
**Comparison of different variants of the precursor charge determination algorithm**. This plot shows ROC curve for range search and choosing different scenarios of the precursor charge assignments in LDA classifier.

Since we identified the features (both precursor peaks and neutral losses) that can classify ETD MS/MS spectra into different precursor charge states, we then compared the differences in ROC plots for different variants of precursor charge assignments. In the 1^st ^OMSSA run, we considered only the best charge (top 1) given by LDA to all the spectra in the test set. For the 2^nd ^run, we considered top 1/top 2 option, where we assigned the best charge to spectra which have posterior probability above a certain threshold t1, while assigning top 2 charge states to the remaining spectra. For the 3^rd ^run, we considered 1/2/All variant. For the 4^th ^run, we considered range search option. Figure 5 shows 1/2/All variant works well compared to the top1 or top1/top2 variants and also performs better over the range search method. Using this LDA model on the training set resulted in the misclassification error rates of 5.14%, 3.03% and 2.33% for the Top 1, Top 1/Top 2 and 1/2/All methods respectively. For the test set, the misclassification error rates were 11.48%, 7.6% and 2.36% for the Top 1, Top 1/Top 2 and 1/2/All methods respectively.

As we mentioned earlier, one of the reasons to determine precursor charge is to reduce the database search time and to decrease the number of false identifications. The computational times for the OMSSA range search and the OMSSA search after precursor charge assignments is shown in Table 1. It can be seen that if we assign only the best charge to all of the spectra in the test set, the search is almost 10 times (91/9) faster than the range search method. However, this results in loss of peptide identifications. Similarly, we lose some true positives with top 1/top 2 option. Since it is important not to lose any identifications, the 1/2/All variant looks optimal and is 3.5 times faster than the range search method.

Apart from the precursor peaks and neutral losses, we also considered the density and distribution of product ions as a feature. The density and distribution of the product ion peaks in ETD spectra depends on the precursor charge i.e., a 3+ precursor charge peptide ion can produce product fragment ions up to 2+ charge and a 4+ precursor charge ion can produce up to 3+ charge. It can be inferred that higher the charge of the precursor, the denser the product ion peaks in the MS/MS scan, although to a small degree this is counteracted by a reduction in the intensity of the charge reduced precursors. This kind of approach was used previously to differentiate between 2+ and 3+ precursor charge states in CAD data [25]. We also used a similar approach to see if there is further increase in sensitivity using product ion distribution as a feature. From our analysis, we could not see any improvement using product ion distribution as another input feature to LDA.

**Figure 6:**
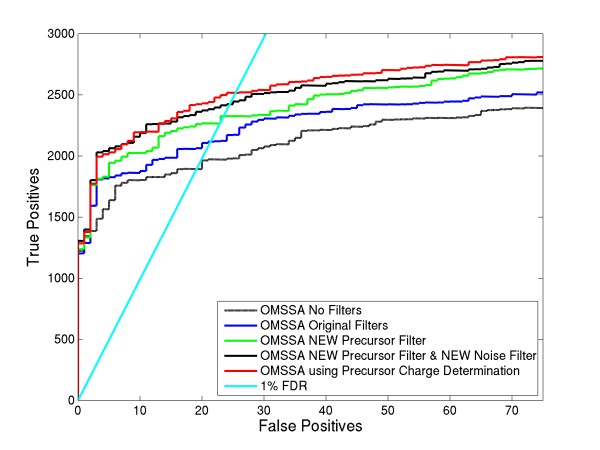
**Combined improvement by updating the precursor filter, noise filter and estimating precursor charge state**. This ROC plot shows the improvement in sensitivity of peptide identifications by employing different kinds of spectral pre-processing steps. Overall, there is a 18.8% increase in identifications at 1% FDR with the new precursor and noise filters and using charge assignment, when compared to the older filters in OMSSA using range search method.

To summarize the effects of these spectral pre-processing steps and precursor charge estimation algorithm, we plotted all the ROC curves in Figure 6. Table 2 shows the improvements in sensitivity with the new modifications to the precursor and the noise filter and with precursor charge determination.

**Table 2:** Table showing sensitivity increase of peptide identifications with updated filters and precursor charge estimation.

OMSSA Search	True positives (1% FDR)	Improvement
Original Filters	2118	--

New Precursor Filter	2326	9.8%

New Precursor and Noise Filters	2423	14.4%

Precursor Charge Determination Algorithm	2516	18.8% (3.5 times faster)

This table shows the number of true positives (TP) at 1% FDR for various stages of spectral processing and precursor charge assignment. A column showing percent increase (over OMSSA with original filters and range search option) in peptide identifications for each method is also provided.

## Conclusion

ETD can dissociate precursor ions over a wide charge range. MS/MS spectra of these peptides can have charge reduced precursor peaks with corresponding neutral losses, all of which are generally intense. To reduce false positives and false negatives, these peaks should be removed before submitting the spectra to a protein database search algorithm for peptide identification. We developed an algorithm to remove these precursor peaks and neutral losses more effectively. We removed bins upstream of precursor peaks of width proportional to the molecular weight of the precursor. Similarly we removed the possible neutral losses associated with these precursor peaks in the ETD spectra. ROC plots (see Figure 2) show better performance compared to the previous OMSSA filter and the spectral pre-processing algorithm developed by Good et. al. [23]. There was an increase of at least 9.8% identifications at 1% FDR when OMSSA's precursor filter is compared to the original OMSSA filter (see Figure 6).

An additional improvement to the spectral pre-processing was based on the observation that ETD spectra can contain different ion-series such as y ions, depending on the precursor charge of the peptide. We incorporated this information into OMSSA's noise filtering. This led to a further 4.2% increase in peptide identifications at 1% FDR (see Figure 6). Charge reduced precursor peak filtering along with the noise filtering should result in increase in peptide identifications for MS/MS data obtained from both the lower resolution and higher resolution instruments. We did not test the filters on data from a high resolution instrument.

Precursor charge is often not measured on lower resolution instruments, although the distribution of charge reduced precursor peaks in the MS/MS spectra has a pattern that determines the precursor charge. In this study, we used this pattern of charge reduced precursor peaks and their neutral losses to determine the precursor charge. Neutral loss peaks can aid in classifying the ambiguous charge states, i.e., multiples such as 3+/6+ etc. We developed an algorithm to predict parent precursor charge state using statistical methods. Using LDA, we determined that the intensity and pattern of charge reduced precursor peaks and neutral losses were found to be a good predictor of the precursor charge. Using this precursor charge determination algorithm, OMSSA's run times were 3.5 times faster compared to range search method, with a minor 3.8% increase in peptide identifications at 1% FDR (see Figure 6). Previous charge determination algorithms did not report any increase in sensitivity of peptide identifications, while our algorithm clearly showed small increase in sensitivity. MS/MS database search algorithms could incorporate charge state determination algorithms as an important tool in significantly reducing database search times. Overall, using the new versions of the precursor and noise filters in OMSSA and incorporating charge determination algorithm, there was an increase of at least 18.8% in peptide identifications and almost 3.5 times faster than the previous version of OMSSA. Such improvement in sensitivity and the database search times with the updated filters and precursor charge determination could be useful for mass spectrometry labs with lower resolution instruments.

## Methods

ETD MS/MS spectra of yeast phosphopeptides is used for this study [21]. These spectra were acquired using the Finnigan LTQ mass spectrometer (Thermo Electron, San Jose, CA). This spectrometer was equipped with a nano-flow HPLC microelectrospray ionization source and was modified to facilitate ETD. The dataset used for this study has a total of 16901 spectra, of which 10000 spectra were used as training set, while the rest were used as test set. We compared the charge state breakdown of the training set and test sets for hits with an e-value better than 1e-6. There were 20.1%, 51.4%, 19.4%, 7.7% and 1.4% peptide hits of +3, +4, +5, +6 and +7 charge states respectively in the training set. In the test set, there were 22.8%, 44.4%, 20.1%, 10.7% and 2.0% peptide hits of +3, +4, +5, +6 and +7 charge states respectively. The charge state distributions are approximately same for both training and test sets. Of the peptide identifications, there were only 18 unique peptide hits that were common to both training and test sets.

The OMSSA precursor and noise filtering algorithm was prepared using the NCBI C++ toolkit. For precursor charge determination, we used LDA, where precursor charge states are the predefined classes. LDA is done using MATLAB 7.8.0 [R2009a]. We wrote scripts in MATLAB to extract features from the spectra to input into LDA.

After the spectral processing is done and precursor charge states assigned for the spectra, we used OMSSA 2.1.7 for peptide identification. Here is a brief outline of the parameters and the sequence library used for the OMSSA search. A static modification of alkylation with iodoacetamide on cysteine, static modifications of methyl ester formation on aspartic acid, glutamic acid and the peptide C terminus, a variable modification of oxygen on methionine and phosphorylation of serine, threonine and tyrosine are considered. A precursor mass tolerance of 3.0 Da, and a fragment mass tolerance of 0.4 Da is used and c, z**˙ **and y ions are searched in these ETD MS/MS spectra. For all our analyses, we searched the MS/MS spectra against a target-decoy library [22] using target sequences from the NCBI 6298 yeast protein sequence library. Using the target-decoy database strategy, we get decoy and the forward database assignments. The number of false positives is generally considered equal to the decoy assignments, while the number of true positives is the forward database assignments minus the decoy database assignments at the e-value considered. OMSSA search results are then analyzed using the receiver operating characteristic [ROC] curves. ROC curve is a plot of sensitivity (true positives) plotted against 1-specificity (false positives). All the OMSSA searches were run on a cluster of SuSe linux machines.

## Competing interests

The authors declare that they have no competing interests.

## Authors' contributions

VS executed the experiments, did the data analysis and wrote the manuscript. DLB and LYG participated in the data analysis and revised the manuscript. AC conducted experiments to obtain yeast phosphoproteome data sets and also helped revise the manuscript. VS and LYG were responsible for design of the experiments. DLB, DFH, JS, SHB and LYG supervised these experiments. All authors have read and approved the final manuscript.
